# Supplementary material for: Enterobacter cloacae infection characteristics and outcomes in battlefield trauma patients
Source: PLoS One. 2023 Aug 29;18(8):e0290735. doi: 10.1371/journal.pone.0290735 (PMC10464967; doi:10.1371/journal.pone.0290735)
Supplement: Attachment — Submitted filename: Response to Reviewers.docx [file pone.0290735.s002.docx]

**RESPONSE TO REVIEWERS**

**ACADEMIC EDITOR:**

*You have done a good job with the suggestions and changes of manuscript.*

**Author Response: Thank you very much.**

I suggest a minor change that implies taking the reviewer's suggestion that I indicate below:

"In figure 1, I suggest to delete nitrofurantoin as the manuscript refers to soft tissue and bone infections. Also, cefuroxime should be deleted as is intrinsically resistant. Ceftaroline is not the preferred drug for E. cloacae. It may be removed also."

Despite the fact that you indicate and justify it as a broad-spectrum antibiogram, in reality clinical microbiology should inform the antibiogram according to the identified microorganism and the site of infection. I think that this figure can be an element of confusion for the reader.

**Author Response: Thank you for your suggestion. Figure 1 has been modified per your comments and ceftaroline has been removed from the sentence on lines 399-403.**

**Journal Requirements:**
Please review your reference list to ensure that it is complete and correct. If you have cited papers that have been retracted, please include the rationale for doing so in the manuscript text, or remove these references and replace them with relevant current references. Any changes to the reference list should be mentioned in the rebuttal letter that accompanies your revised manuscript. If you need to cite a retracted article, indicate the article’s retracted status in the References list and also include a citation and full reference for the retraction notice.

**Author Response: Thank you. The reference list has been reviewed and it is complete. There was a change for the URL for reference #19, which was updated in the revised version. Reference #26 was also updated to reflect the current version available for that document. The rest of the references are correct. None of the papers cited have been retracted.**
